# Obstetric and maternal outcomes of IVF and oocyte donation pregnancies among women ages 40–45—a large cohort study

**DOI:** 10.1007/s10815-024-03094-w

**Published:** 2024-03-23

**Authors:** Roni Rahav-Koren, Hila Shalev-Ram, Einat Haikin-Herzberger, Mattan Levi, Amir Wiser, Netanella Miller

**Affiliations:** 1 Department of Obstetrics and Gynecology, IVF Unit, Meir Medical Center, 59 Tchernichovsky St, 4428164 Kfar Saba, Israel; 2 Faculty of Medicine, Tel Aviv University, Tel Aviv, Israel; 3 Department of Obstetrics and Gynecology, Mayanei Hayeshua Medical Center, Bnei Brak, 51544, Israel

**Keywords:** In vitro fertilization, Oocyte donation, Preterm labor, Small for gestational age

## Abstract

**Purpose:**

To analyze the perinatal and maternal outcomes of women ranging in age from 40 to 45 years who gave birth after in vitro fertilization or oocyte donation, compared to spontaneous conception.

**Methods:**

This retrospective cohort study used electronic data from a national healthcare service from 2000 through 2019. Three groups were compared: spontaneous pregnancy (SC), in vitro fertilization (IVF) utilizing autologous oocytes, and pregnancies resulting from oocyte donation (OD). The primary study outcomes were preterm labor (PTL) before 37 weeks of gestation, and infants classified as small for gestational age (SGA).

**Results:**

The cohort included 26,379 SC, 2237 IVF pregnancies, and 300 OD pregnancies for women ages 40–45 years at delivery. Women with OD or IVF had a higher incidence of PTL < 37 weeks compared to women with SC (19.7% vs. 18% vs. 6.9%, *p* = 0.001), PTL < 34 (7% vs. 4.5% vs. 1.4%, *p* = 0.001), PTL < 32 (3.7 vs. 2.1 vs. 0.6, *p* = 0.001). A multivariable logistic regression for PTL < 37 weeks demonstrated that age (OR = 1.18) and hypertensive diseases (OR = 3.4) were statistically significant factors. The OD group had a lower rate of SGA compared to SC (1% vs. 4.3%, *p* = 0.001), while the IVF group had a higher rate of SGA compared to SC (9.1% vs. 4.3%, *p* = 0.001). Hypertensive diseases in pregnancy were significantly higher among the OD group and the IVF group compared to SP pregnancies (3.3% vs. 1%, *p* = 0.002; 2.3% vs. 1%, *p* = 0.001, respectively).

**Conclusions:**

Women ages 40–45 undergoing IVF or OD have a greater risk of PTL, possibly due to higher rates of hypertensive disorders of pregnancy.

## Introduction

Advancements in assisted reproductive technologies (ART) have provided a clinical solution for couples and individuals facing challenges with fertility. ART has gained significant traction, contributing to 2–5% of live births in the USA [1]. Among these ART options, IVF and OD have gained prominence with clinical pregnancy rate of 30–55% for autologous IVF [2, 3] and 40–55% for donated oocytes [2, 4].

A comprehensive understanding of the obstetrical and neonatal outcomes of pregnancies resulting from either autologous or donated oocytes is clinically important. Research studies comparing IVF and SC have revealed a higher prevalence of adverse outcomes, including preterm delivery, low birth weight, very low birth weight, and small for gestational age infants [5, 6]. Similarly, studies comparing OD to SC have shown that singleton pregnancies following OD are associated with significantly increased risks for low birth weight (< 2500 g) infants, preterm labor (PTL) (< 37 weeks of gestation) [7, 8] and higher risk of pre-eclampsia [9].

However, it remains challenging to disentangle the influence of the mode of conception from other critical factors that may contribute to these outcomes. One such confounding factor is maternal age, as OD and occasionally IVF with an autologous oocyte are often pursued by older women who have experienced age-related declines in fertility. Pregnancies at advanced maternal age are associated with adverse obstetrical outcomes, including higher rates of cesarean section, pre-eclampsia, and gestational diabetes mellitus [10–12]. With advanced maternal age and the use of assisted reproductive technologies, it is not clear which factors contribute to the varying obstetric and maternal outcomes of these pregnancies.

The aim of this study was to contribute to clarifying this complex relationship by analyzing perinatal and maternal outcomes within the age range of 40–45 years. By focusing on a large cohort of women in this specific age group, we aimed to minimize the confounding effects of age, allowing us to focus on whether the mode of conception independently influenced obstetric outcomes.

## Methods

### Study design

This retrospective cohort study utilized data from the Maccabi Healthcare Services data registry, the second largest integrated healthcare organization in Israel. The study collected and analyzed electronic medical record data from 2000 to 2019. Prior to analysis, all data were anonymized. Diagnoses were established according to the International Classification of Diseases, 9th Revision, Clinical Modification (ICD-9-CM) criteria.

### Study population

The study cohort was stratified into three groups based on the mode of conception: spontaneous pregnancy (SC), in vitro fertilization (IVF) utilizing autologous oocytes, and pregnancies resulting from oocyte donation (OD). Inclusion criteria comprised singleton pregnancies for women ages 40 to 45 years at the time of delivery. The maximum age of 45 was chosen because IVF procedures are funded in Israel until this age. Exclusion criteria encompassed pregnancies of unknown conception mode, as well as miscarriages and deliveries prior to 24 weeks of gestation. Multiple gestations were also excluded, as were cases lacking well-documented data concerning neonatal gestational age and birth weight.

### Data collection and study outcomes

The collected data included the following:

1. Maternal demographics (age, body mass index (BMI), marital status, and socioeconomic status (SES). SES is calculated based on demography, education, standard of living, and employment and assessed according to the poverty index of the patient’s address enumeration area as defined by the national census statistics. This evaluation yields a SES score on a scale that starts at 1, indicating the lowest socioeconomic status, and extends to 20, representing the highest possible status.
2. Perinatal outcomes (gestational age at birth, birth weight, and neonatal sex).
3. Maternal outcomes (hypertensive diseases in pregnancy (HDP) including gestational hypertension, pre-eclampsia, and chronic hypertension; diabetes mellitus/gestational diabetes mellitus (DM/GDM); malpresentation; and retained placenta.

The primary study outcomes were preterm labor (PTL) defined as birth occurring before 37 weeks of gestation, and infants classified as small for gestational age (SGA). SGA was determined using the 10th percentile threshold derived from Dolberg’s birthweight curves specific to singletons [13].

### Statistical analysis

Data were analyzed using SPSS 24.0 package for windows (SPSS, Inc.) and Phyton 3.7.9. Discrete variables are presented as number and percentage and continuous variables as mean and standard deviation. Student’s *t*-test or *χ*^2^ test was used for continuous and categorical variables, respectively.

Multivariable logistic regression analyses were performed to estimate the risks of PTL < 37 and SGA. The analyses were adjusted for age, BMI, SES, HDP, and GDM/diabetes. For *t*-tests, adjusted *p*-values for three comparisons < 0.016 were considered significant, and for the multivariate regression, *p*-values of < 0.05 were considered significant.

### Ethics approval

The study was approved by the Institutional Review Board of Maccabi Healthcare Services. The requirement for informed consent was waived.

## Results

The cohort included 26,379 SC, 2237 IVF pregnancies, and 300 OD pregnancies for women ages 40 to 45 years at delivery. Data regarding basic characteristics are shown in Table 1. Maternal age was significantly older for the OD pregnancies compared to the IVF and SP group (*p* = 0.001). However, the difference was less than a year. The OD group had lower BMI compared to IVF and SC (25.6 vs. 26.8 vs. 26.5, *p* = 0.004 and *p* = 0.009, respectively); however, the difference was less than 1 year.

**Table 1:** Maternal characteristics of women after OD, IVF, and SC (maternal age 40–45 years)

Characteristic	OD	IVF	SC	*p*-value, OD vs. IVF	*p*-value, OD vs. SC	*p*-value, IVF vs. SC
*N*	300	2237	26,379			
Maternal age, years, mean (SD)	42.3 (1.4)	41.4 (1.3)	41 (1.2)	**0.001**	**0.001**	**0.001**
Body mass index, mean (SD)	25.6 (5.3)	26.8 (6.2)	26.5 (5.6)	**0.004**	**0.009**	**0.079**
Socioeconomic status, mean (SD)	7.2 (1.5)	7.1 (1.7)	6 (2)	0.411	**0.001**	**0.001**
Single, *n* (%)	166 (55.3)	1199 (53.6)	8652 (32.8)	0.582	**0.001**	**0.001**
Parity, mean (SD)	0.37 (0.5)	0.5 (0.6)	1 (1.3)	**0.015**	**0.001**	**0.001**

The OD group had the highest SES compared to SC (7.2 vs. 6, *p* = 0.001) with no difference compared to the IVF group (7.2 vs. 7.1, *p* = 0.411). The prevalence of single women was higher in the OD group compared to the SC group (55% vs. 32%, *p* = 0.001). Parity was significantly higher in the SC compared to the OD and IVF (*p* = 0.001).

Table 2 demonstrates obstetrical and maternal outcomes. Women with OD and IVF had a significantly higher incidence of PTL < 37 weeks compared to women with SC (19.7% vs. 18% vs. 6.9%, *p* = 0.001), PTL < 34 (7% vs. 4.5% vs. 1.4%, *p* = 0.001), and PTL < 32 (3.7 vs. 2.1 vs. 0.6, *p* = 0.001). There was no difference between OD and IVF pregnancies regarding PTL < 37 (*p* = 0.536), PTL < 34 (*p* = 0.059), and PTL < 32 (*p* = 0.101). A lower incidence of SGA was found in the OD group compared to the SC (1% vs. 4.3%, *p* = 0.001), while the IVF group had a higher rate of SGA compared to SC (9.1% vs. 4.3%, *p* = 0.001). HDP was significantly higher in the OD group and the IVF group compared to SP pregnancies (3.3% vs. 1%, *p* = 0.002 and 2.3% vs. 1%, *p* = 0.001, respectively). No difference was found regarding DM/GDM or malpresentation when comparing OD to SC (*p* = 0.146 and *p* = 0.296, respectively). However, a higher prevalence of DM/GDM was found in the IVF group compared to the SC group (2.2% vs. 1.1%, *p* = 0.001). The OD and the IVF groups were comparable in the rates of HDP, DM/GDM, and malpresentation.

**Table 2:** Perinatal outcomes of children born after OD, IVF, and SC (maternal age 40–45)

Characteristic	OD	IVF	SC	*p*-value, OD vs. IVF	*p*-value, OD vs. SC	*p*-value, IVF vs. SC
*N*	300	2237	26,379			
GW, mean (SD)	37 (2.6)	37 (2.1)	39 (1.8)	0.82	**0.001**	**0.001**
GW < 37, *n* (%)	59 (19.7)	402 (18)	1820 (6.9)	0.536	**0.001**	**0.001**
GW < 34, *n* (%)	21 (7)	100 (4.5)	369 (1.4)	0.059	**0.001**	**0.001**
GW < 32, *n* (%)	11 (3.7)	47 (2.1)	158 (0.6)	0.101	**0.001**	**0.001**
Birth weight, grams; mean (SD)	2992 (642)	2972 (607)	3271 (523)	0.602	**0.001**	**0.001**
SGA, *n* (%)	3 (1)	203 (9.1)	1135 (4.3)	0.966	**0.001**	**0.001**
LGA, *n* (%)	33 (11.3)	210 (9.4)	4141 (15.7)	0.295	0.039	**0.001**
Female sex, *n* (%)	46 (48.7)	1120 (50.2)	13,532 (51.3)	0.608	0.364	0.337
HDP, *n* (%)	10 (3.3)	51 (2.3)	263 (1)	0.288	**0.002**	**0.001**
DM/GDM, *n* (%)	6 (2)	49 (2.2)	290 (1.1)	0.795	0.146	**0.001**
Malpresentation, *n* (%)	3 (1)	38 (1.7)	131 (0.5)	0.344	0.296	**0.001**
Retained placenta, *n* (%)	0 (0)	2 (0.1)	26 (0.1)	0.526	0.67	0.195

*GW* gestational week, *LGA* large for gestational age above 90th percentile, *SGA* small for gestational age below 10th percentile, *HDP* hypertensive disorders of pregnancy (pre-eclampsia, eclampsia, gestational hypertension), *DM* diabetes mellitus, *GDM* gestational diabetes mellitus

A multivariable logistic regression for PTL < 37 weeks demonstrated that age (OR = 1.18) and HDP (OR = 3.4) were statistically significant factors (Table 3). OD and IVF were not significant factors.

**Table 3:** Multivariable logistic regression analysis for preterm birth < 37 weeks

Variable	Odds ratio	95% CI for OR		*p*-value
		Lower	Upper	
Maternal age, years	1.18	1.1	1.26	**0.001**
Body mass index	0.96	0.93	1	0.063
Socioeconomic status	0.96	0.87	1.06	0.45
Parity, mean (SD)	0.95	0.77	1.18	0.695
IVF	1.95	0.65	5.8	0.227
OD	1.01	0.7	1.45	0.951
HDP (pre-eclampsia, eclampsia, gestational hypertension)	3.4	1.3	8.2	**0.007**
Diabetes (DM, GDM)	0.99	0.38	2.6	**0.998**

*HDP* hypertensive disorders of pregnancy, *DM* diabetes mellitus, *GDM* gestational diabetes mellitus

A multivariate logistic regression for SGA demonstrated that maternal age (OR = 1.09), BMI (OR = 1.2), and OD (OR = 0.49) were significant factors (Table 4). Maternal age was not found to be a factor for PTL < 34, nor for HDP.

**Table 4:** Multivariable logistic regression analysis for SGA

Variable	Odds ratio	95% CI for OR		*p*-value
		Lower	Upper	
Maternal age, years	1.09	1	1.2	**0.045**
Body mass index	0.93	0.88	0.98	**0.010**
Socioeconomic status	0.9	0.78	1.03	0.146
Parity, mean (SD)	0.83	0.6	1.14	0.26
IVF	0.86	0.1	6.9	0.889
OD	0.49	0.25	0.99	**0.046**
HDP (pre-eclampsia, eclampsia, gestational hypertension)	1.68	0.42	6.6	0.455
Diabetes (DM, GDM)	0.72	0.14	3.6	0.697

*HDP* hypertensive disorders of pregnancy, *SGA* small for gestational age, *DM* diabetes mellitus, *GDM* gestational diabetes mellitus

## Discussion

This study’s findings indicate that women aged 40 to 45 who undergo OD or IVF have a significantly higher risk of experiencing preterm labor before 37, 34, and 32 weeks of gestation, in comparison to those who conceive spontaneously. The data suggests that maternal age and, particularly, hypertensive disorders of pregnancy (HDP) are the primary factors influencing the incidence of preterm labor (PTL), with IVF and OD being surrogate markers.

This study also shows that IVF is also associated with a higher incidence of SGA neonates, whereas OD tends to have a reduced occurrence of SGA compared to SC.

Our findings support those of previous studies that demonstrated a higher rate of preterm labor (PTL) in pregnancies after IVF and OD conceptions. A meta-analysis that included 15 studies showed that singleton IVF pregnancies have a higher rate of PTL compared to SC (aOR 1.95), after controlling for confounders of maternal age and parity [5]. Yet most studies in this meta-analysis did not address the effect of HDP. A second meta-analysis of 20 studies revealed a relative risk of 1.54 having delivery before 37 weeks in IVF/ICSI conceptions, when compared with SC [6]. This analysis also showed a relative risk of having hypertensive disorder of pregnancy in IVF/ICSI conceptions, when compared with SC.

Data regarding OD also show a higher rate of PTL. A study investigating 375 children born after OD showed an aOR of 1.8 for PTL compared to SC [9]. A meta-analysis also found an aOR of 2.3 for PTL of OD pregnancies vs. SC, also adjusting for maternal age [14].

The higher rate of PTL in these pregnancies can be attributed to several factors: (1) maternal age and its related characteristics such as BMI and SES, (2) the use of reproductive technology in IVF and OD, (3) diseases that might contribute to PTL could be an underlying cause of infertility, (4) using OD, and (5) higher rates of HDP or DM/GDM that may be related to a higher rate of iatrogenic PTL.

This study sought to control for maternal age by investigating only women ages 40–45, thereby reducing its influence as a variable. Moreover, data regarding advanced maternal age and PTL are still controversial. Some studies reported a higher rate of PTL for women of advanced maternal age [10, 15]; yet, another did not find a definite association [16]. Based on the adjusted analysis for age, we found that maternal age still contributed to PTL (OR 1.18), even between the range of 40–45 years.

Concerning the intricacies of the IVF process, including the administration of hormones and the manipulation of gametes and embryos in the laboratory, it could be hypothesized that these interventions influence the onset of PTL. The observation that both the IVF and OD cohorts exhibited an increased prevalence of PTL in comparison to those conceiving naturally suggests a potential association. Furthermore, the absence of a significant difference in PTL rates between the IVF and OD groups lends weight to this hypothesis. This conclusion also applies to the explanation that underlying diseases that may cause PTL could contribute to infertility. Yet, in the adjusted analysis, we did not find effects of IVF or OD on PTL, which may suggest that IVF and OD may be related to PTL indirectly, encompassing other factors that may contribute to PTL, such as age and HDP.

OD alone may contribute to PTL, as has been shown in some studies [9, 14]. One explanation is that OD pregnancies have a higher incidence of HDP compared to IVF and SC. A meta-analysis analyzing 28 publications found an odds ratio for HDP after OD of 2.57 compared to IVF and an odds ratio of 6.60 compared to SC pregnancies [17]. A systematic review and meta-analysis based on 19 studies also demonstrated that OD increased the risk of pre-eclampsia and gestational hypertension, compared with other ART methods or SC [18]. Savasi et al. [19] also found higher rates of placental disorders of pregnancy, such as gestational hypertension and pre-eclampsia in OD pregnancies. A higher rate of HDP was found regardless of maternal age. In addition, the Swedish study which analyzed 375 children born following OD indicated an augmented risk of pre-eclampsia in OD pregnancies, with an adjusted odds ratio (AOR) of 3.1 in comparison to spontaneous conception (SC). Keegan et al. [20] found that even women younger than 35 receiving OD had a high rate of HDP compared to older IVF patients. Shefller-Mimoini [21] also demonstrated a higher rate of HDP in OD pregnancies. Their analysis showed that advanced maternal age was not an independent risk factor for hypertensive disorders in pregnancy. A possible physiological explanation is related to the totally allogenic transplant, since the fetal antigens are 100% different from the mother’s, which may cause an abnormal response and play a role in the pathogenic mechanism of hypertensive diseases in these pregnancies [19, 22]. Also, our multivariable analysis showed that hypertension itself is a significant factor in PTL, suggesting that OD may be a risk factor for PTL indirectly, through the higher incidence of HDP in these pregnancies.

Our findings showed that IVF pregnancies have a higher incidence of SGA, whereas OD has a lower rate of SGA. Studies comparing IVF to SC have reported higher rates of SGA as shown in a review by Bernsten et al. [23]. An Australian data linkage cohort found an increased risk for SGA births with adjusted risks of approximately 1.5 among children born after IVF vs. SC [24]. This may be because IVF pregnancies have more obstetric complications, such as hypertensive disorders and placental complications (placenta previa, abruption, and third trimester bleeding), which may contribute to SGA neonates [23].

If the presence of SGA infants is associated with HDP, then one would anticipate a higher incidence of SGA within the OD group, particularly given the increased prevalence of HDP in these cases. However, contrary to previous research that suggests OD pregnancies have a higher SGA rate when compared to SC [7, 19, 21, 25], our data indicate a notably lower incidence of SGA in OD pregnancies.

This discrepancy may be attributed to the prevalent use of frozen embryo transfer (FET) cycles for OD cycles that involve artificial endometrial preparation using estradiol and progesterone in our region, as opposed to fresh cycles which are more common for IVF with autologous oocyte cycles. Recent studies indicate that such FET pregnancies, based on artificial endometrial preparation, do not exhibit an increased risk of SGA, but rather a tendency toward large for gestational age infants, possibly due to the absence of the corpus luteum [26].

The data from this cohort indicate that IVF and OD pregnancies in women ages 40–45 years are associated with a higher incidence of PTL, which might be related to maternal age and to increased rate of HDP. These findings imply the need for increased monitoring of these women during pregnancy, potentially including prophylactic use of low-dose aspirin to reduce the risk of HDP and meticulous screening for cervical length to forestall PTL.

The advantages of this study are derived from its relatively large sample size and the tripartite comparative analysis encompassing IVF, OD, and SC cohorts. The study is also bolstered by the inclusion of women in the 40–45 age range only, establishing age as a fixed variable. This enabled a focus on alternative factors that might influence obstetric and maternal outcomes in these pregnancies.

Yet, this study is not without limitations. In a retrospective analysis with large datasets, there is an acknowledged underreporting of data, specifically within the scope of the ICD-9 classifications pertaining to obstetrical complications, including HDP and DM/GDM. The missing high-risk diagnosis data stemmed from instances where the diagnosing physician did not document the condition, making it challenging to ascertain the exact extent of missing information. For this reason, missing data could not be handled. We acknowledge this limitation and posit that any potential underdiagnosis is likely to be uniformly distributed across all study groups, which would mitigate systematic bias in the comparative analysis. Additionally, the study lacked comprehensive details regarding the protocols employed for embryo transfer, which may have helped reinforce our findings. Also, data on whether the PTL was induced or spontaneous is lacking. Further prospective and retrospective studies should be conducted to reevaluate our finding.

## Conclusion

Women ages 40–45 undergoing IVF or OD have a greater risk of PTL, possibly due to higher rates of HDP. These findings highlight the need for tailored pregnancy management in these patients, possibly including low-dose aspirin to prevent HDP and cervical length monitoring to mitigate the risk of PTL risk.

## Data Availability

Data cannot be shared since it is the property of Maccabi Health Services.
